# Preterm Birth, Low Gestational Age, Low Birth Weight, Parity, and Other Determinants of Breech Presentation: Results from a Large Retrospective Population-Based Study

**DOI:** 10.1155/2019/9581439

**Published:** 2019-09-16

**Authors:** Stefania Antonia Noli, Ilaria Baini, Fabio Parazzini, Paola Agnese Mauri, Michele Vignali, Sandro Gerli, Alessandro Favilli, Sonia Cipriani

**Affiliations:** ^1^Dept of Clinical Sciences and Community Health, Università degli Studi di Milano, Milan, Italy; ^2^IRCCS Fondazione Ca' Granda, Ospedale Maggiore Policlinico, Milan, Italy; ^3^Obstetrics and Gynecology Unit, P.O. Macedonio Melloni-ASST Fatebenefratelli-Sacco, Milan, Italy; ^4^Dept of Biomedical Sciences for Health, Università degli Studi di Milano, Milan, Italy; ^5^Department of Obstetrics and Gynecology, University of Perugia, S.M. Della Misericordia Hospital, Perugia 06156, Italy

## Abstract

Aim of this study is to analyze determinants of breech presentation using information from two regional databases of Lombardy (Italy) including data on consecutive singleton breech and vertex deliveries occurred in the Region, between January 2010 and December 2015. Breech presentation occurred in 3.8% of all single deliveries. Main determinants of breech presentation at birth were: gestational age and birth weight (the lower, the higher the incidence of breech presentation), maternal age (the older the mother, the higher the risk of breech presentation), parity (the frequency of breech decreased with increasing parity) and previous cesarean section. Breech presentation resulted more frequent after assisted reproduction procedures.

## 1. Introduction

The most common cause of breech presentation is preterm delivery [1]. Other commonly suggested determinants are fetal growth retardation and oligo or polyhydramnios [2–4].

Among maternal characteristics, older age, primiparity, and previous cesarean section have been suggested as risk factors for breech delivery, but their role is still controversial.

Only few population-based studies have been published on risk factors for breech presentation [1, 5, 6] and, particularly, few data are available from Italy and South European countries, regions presenting some epidemiological peculiarities. They are actually characterized by different reproductive patterns and by higher rates of cesarean section. In this paper, we have investigated the risk factors for breech presentation using data derived from a large data set from Lombardy, a high populated Region in Italy.

## 2. Methods

In Lombardy (an Italian region of about 10 million inhabitants), a standard form is used to register all births and neonatal discharges from public and private hospitals. Diagnoses of admission and discharge are codified according to the International Statistical Classification of Diseases 9th edition—Clinical Modification (ICD-9-CM), Italian version. For each delivery, both maternal and neonatal demographic and clinical data are reported (i.e., maternal country of birth, maternal cause of admission, neonatal sex, congenital abnormalities detected at birth or within the period of hospital admission). After delivery, a specific form is filled by midwifes including information on current pregnancy and its clinical course, on maternal characteristics and medical history, on type of conception (spontaneous/not spontaneous), on delivery (including fetus presentation), and on maternal outcome at birth.

Data on all deliveries and births in Lombardy from January 2010 to December 2013 and from January 2014 to December 2015 were included in the present analysis; multiple gestations were excluded from the analysis as they are characterized by significantly increased risk of noncephalic presentation; deliveries with transverse and oblique presentation were excluded.

Comparisons have been made between breech deliveries and vertex deliveries. Potential risk factors have been chosen based on previous literature. Crude Odds ratios (OR) with corresponding 95% confidence intervals (CI) were computed. Further, multivariate estimates, derived using unconditional logistic regression, fitted by the method of maximum likelihood, were also computed. Small for gestational age (SGA) at birth was defined according to previous publications [7, 8].

According to Italian law, analysis of anonymous routine database do not require Ethics Committee application.

## 3. Results

In the study period 450,104 deliveries were reported. After exclusion of stillbirths (493), twins (14346), newborns with gestational age <23 weeks or missing (537), and presentation different from vertex and breech (*n* = 1906), 432,822 deliveries were considered in our analysis. Breech presentation occurred in 16,525 (3.8%) cases.

Table 1 shows in detail the results of our analysis. In particular, the risk of breech delivery decreased with increasing gestational age at birth (OR = 16.08, 95% CI 12.18–21.22 and OR = 2.68, 95% CI 2.52–2.84 for, respectively 23-24 and 35-36 weeks of gestation, compared to 37 weeks of gestation or more), while it significantly increased with maternal age.

The risk of breech presentation was lower in parous women: the adjusted OR was 0.47 (95% CI 0.45–0.49) for women with one previous delivery and 0.40 (95% CI 0.37–0.43) for women with two or more previous deliveries compared to nulliparous women.

Breech presentation was significantly associated with a history of previous cesarean section (OR = 1.81, 95% CI 1.70–1.93, for women who had one previous cesarean delivery compared to parous women with no previous CS) and it was also related to assisted reproduction technology conceptions (adjusted OR 1.14, 95% CI 1.04–1.25), previous spontaneous/induced abortion (OR = 1.05, 95% CI 1.01–1.09), female sex (adjusted OR = 1.22, 95% CI 1.18–1.26), and to SGA (small for gestational age) births (OR = 1.34, 95% CI 1.27–1.40).

Further, we conducted the same analysis in the subset of both vertex and breech births occurring at 37 weeks of gestation or more (Table 2). The results of this subgroup analysis are basically consistent with those referring to all deliveries at any gestational age and, in particular, breech birth at 37 weeks or more was still significantly associated with SGA births (OR = 1.25, 95% CI 1.19–1.32). On the contrary, no significant association was observed between the risk of breech delivery after 37 weeks and a history of two or more previous cesarean sections, compared to parous women without previous CS (OR = 1.60, 95% CI 1.40–1.82). Similarly, the association with assisted reproduction technology conceptions and with a history of previous abortion (spontaneous/induced) totally disappeared.

## 4. Discussion

In this study, the main factors associated with a higher risk of breech presentation were: lower gestational age, low birth weight (SGA newborn), increasing maternal age, primiparity, a history of previous caesarean section, pregnancy after ART procedures, a history of previous spontaneous/induced abortion, and female sex. Most of these conditions remained significantly associated to breech delivery also in the subgroup of births at 37 weeks of gestation or more.

In particular, low birth weight, low gestational age, advanced maternal age, primiparity and a female baby emerged as independent risk factors for breech delivery in several previous papers [1–3, 9, 10]; on the contrary, for some other conditions, inconsistent results were described. Differently from our data and from the analysis by Cammu et al. [1], no specific association between previous cesarean section and breech presentation was observed in the study by Frucalzo et al. [3]. Moreover, additional risk factors for breech delivery, not included in our analysis, emerged in the above-mentioned studies, such as the presence of congenital malformations [1], decreased intra-amniotic fluid [9], or maternal smoking during pregnancy [2, 9].

Before discussing the results, potential limitations of our study should be considered.

Fetal position at delivery and relative risk factors were usually derived from birth and/or hospital records collected mainly for administrative reasons and not for scientific purposes. However, fetal presentation is an easy information to collect and it has a notable relevance in the indication, for example, of cesarean section. Further, the reported frequency of breech presentation was largely consistent during the study period and with data reported for other Italian Regions and World Countries [2, 3, 6]. In any case, misclassification of fetal presentation should tend to underestimate the associations emerging in our study.

Another limitation of this paper is that we have no information about factors such as maternal uterine anomalies, recurrence risk of breech presentation, and hereditary predisposition to breech presentation at birth: all these conditions are actually associated with an increased risk of breech presentation [11].

Among the strengths of this study, we should consider the population-based design and the large sample size. The large number of records gave us the possibility to adjust for several risk factors and it allowed more precise risk estimates. Moreover, to our knowledge, this is the first population-based contribution on this topic coming from Southern Europe and referring to a recent historical period.

Apart from preterm birth, it has been shown that the risk of breech presentation is associated with lower parity and increasing maternal age. In the study by Wiktop et al., a prenatal ultrasound was performed in more than 7000 pregnant patients and they observed that a noncephalic fetus at 35 weeks, in nulliparous women, had twice the chance of not turning in vertex position at delivery, compared to multiparous patients [10]. Rayl et al. observed a decrease of almost 40% in the risk of breech presentation with increasing parity [2]. We confirm these findings: the more extensible uterine and abdominal wall, characterizing multiparous women, probably make easier the spontaneous cephalic version of the fetus close to term and it may represent an explanation of the observed association [1, 3].

A history of cesarean section represents a risk factor for several adverse obstetric outcomes and it has already been described as a predicting condition for breech presentation [5, 6]. Cammu et al. observed a 44% increased risk of breech in women with a previous cesarean section [1]. It is well established that breech presentation tends to recur in subsequent pregnancies of a woman with a previous fetus in breech position [12]. Moreover, taking into account that about 70–85% of all breech infants are delivered by cesarean section, just the presence of a uterine scar might not explain the increased risk of breech presentation [1]. As in most other studies, the indication for a previous cesarean section was not reported in our databases [5, 6].

An interesting finding of our analysis is the observation that breech presentation occurs more frequently in ART pregnancies. This association has been previously reported [1, 6, 12, 13]; however, it is still not clear if this increased risk is caused by the technology itself or it can be attributed to other conditions associated with assisted fertilization. Actually, women who undergo ART, tend to be older, nulliparous and to have shorter gestations: all these factors can explain the higher crude risk of breech delivery associated with ART. Romundstad et al. reported that the crude risk was significantly higher in ART than in spontaneous conceptions, but after adjustment for maternal age, parity, and length of gestation, the difference between the two subgroups was completely attenuated [12]; the same observation was reported in the study by Cammu et al. [1]. Similarly, in our analysis, this association tended to lower after taking into account potential covariates, being the crude estimated OR 1.8, and it completely disappeared when the analysis is conducted in the subgroup of deliveries after 37 weeks.

Our analysis showed that the risk of breech position is significantly higher in SGA children and that small fetal size at birth represents a risk factor independently of gestational age. This result was confirmed in the subgroup analysis of breech birth after 37 weeks. Moreover, the fact that most breech births (>80%) occurs at term supports our observation [2]. Several studies described conditions associated with both small fetal size and breech presentation and they reported the same association between birth weight and breech position after adjustment for gestational age [1–3, 6]. In particular, in the work by Roberts et al. [14], they used birth weight percentiles by gestational age to control for the effect of gestational age on fetal size and they analyze the association between infant weight and breech birth. Their results showed that the risk of breech birth consistently increased as the infant birth weight percentile lowered; similarly, the odds of breech position increased with maternal age, female sex, primiparous birth and the presence of congenital anomalies: a subgroup of conditions generally characterized by smaller infant size at birth [14, 15]. Therefore, the observed increase in the risk of breech birth may be an effect of weight, as well.

Different hypothesis have been so far proposed to clarify this consistent association between fetal size and breech position. One explanation is that some smaller fetuses might have a degree of intrauterine growth restriction (IUGR) and, in this situation, a low amniotic fluid amount or a less active fetus may reduce the chance of turning into cephalic position; however, birth weight and breech birth remain inversely associated up to the 90^th^percentile and a very few of these fetuses would have a IUGR. Another explanation is that a higher fetal weight, mainly distributed to fetal head, forced the fetus into cephalic position in uterus and it may help a spontaneous vertex version [2, 3], while smaller fetuses may be more prone to changing their in-utero presentation. These observations lead to the possibility that the relationship between preterm birth and breech birth is mainly a consequence of smaller fetal size of preterm babies. Hence, increasing duration of pregnancy may allow breech presenting fetuses more time to grow and improve their chances of turning either spontaneously or by external cephalic version (ECV) and to remain in cephalic position [14]. Results of different previous trials support this conclusion as they show that between 15% and 33% of fetuses, that are breech at term (37 weeks), will spontaneously turn to a cephalic position [4, 16]. Unfortunately, the databases we used for our analysis do not include information about the amount of breech presenting fetus at term (they only reported data on breech births), the number of ECVs practiced and their success rate.

In conclusion, this study showed that breech presentation is associated with several maternal and fetal characteristics. Our results are in line with previous reported data but with the peculiarity of being referred to a large specific population in Southern Europe (Lombardy, Italy). These findings suggest that different, and still not well-defined, biologic mechanisms may be involved in determining breech position, including both genetic and acquired environmental factors. Due to the large sample size, this population-based study offers quantitative estimates of the role of different risk factors with narrow confidence intervals.

## Figures and Tables

**Table 1:** Distribution and corresponding odds ratios of cases with vertex and breech presentation according to selected factors. Lombardy, Italy.

	*Presentation*				*Unadjusted logistic regression*			*Adjusted logistic regression *∗		
	*Vertix*		*Breech*			*95% CI*			*95% CI*	
	*N*	*%*	*N*	*%*	*OR*	*Lower*	*Upper*	*OR*	*Lower*	*Upper*
*Maternal age*										
<25	43408	97.0	1344	3.0	1+			1+		
25–29	90850	96.6	3165	3.4	1.12	1.05	1.20	1.23	1.15	1.31
30–34	140980	96.1	5750	3.9	1.32	1.24	1.40	1.50	1.41	1.60
35–39	110433	95.9	4763	4.1	1.39	1.31	1.48	1.67	1.56	1.79
40+	29461	95.3	1448	4.7	1.59	1.47	1.71	1.84	1.69	2.00
*Maternal education*										
University	118047	96.1	4844	3.9	1+			1+		
High school degree	179993	96.1	7367	3.9	1.00	0.96	1.04	1.08	1.04	1.12
Primary/intermediate school or no education	116464	96.5	4247	3.5	0.89	0.85	0.93	1.09	1.04	1.14
*Citizenship*										
Italian	293243	95.9	12379	4.1	1+			1+		
Foreign	123054	96.7	4146	3.3	0.80	0.77	0.83	0.95	0.91	0.99
Albanian	11036	96.6	388	3.4	0.83	0.75	0.92			
Chinese	6277	97.5	163	2.5	0.62	0.53	0.72			
Egyptian	7189	97.2	205	2.8	0.68	0.59	0.78			
Moroccan	16206	97.1	478	2.9	0.70	0.64	0.77			
Romanian	14371	95.8	623	4.2	1.03	0.95	1.11			
Other	67975	96.7	2289	3.3	0.80	0.76	0.83			
*Previous delivery*										
0	210014	95.2	10689	4.8	1+			1+		
1	147313	97.2	4239	2.8	0.57	0.55	0.59	0.47	0.45	0.49
2+	51874	97.5	1311	2.5	0.50	0.47	0.53	0.40	0.37	0.43
Previous parity in unidentified number	6932	96.1	282	3.9	0.80	0.71	0.90	0.55	0.48	0.63
*Previous caesarean*										
Nulliparous	210014	95.2	10689	4.8	2.15	2.07	2.24	NE		
0	133385	97.7	3156	2.3	1+			1+		
1	38316	95.5	1821	4.5	2.01	1.89	2.13	1.81	1.70	1.93
2+	7808	96.4	291	3.6	1.58	1.39	1.78	1.60	1.40	1.82
*Previous spontaneous or induced abortion*										
No	294791	96.1	11849	3.9	1+			1+		
Yes	121506	96.3	4676	3.7	0.96	0.92	0.99	1.05	1.01	1.09
*Mode of conception*										
Spontaneous conception	408332	96.2	15959	3.8	1+			1+		
Assisted conception	7965	93.4	566	6.6	1.82	1.67	1.98	1.14	1.04	1.25
*Gestational age (weeks)*										
23-24	176	69.0	79	31.0	12.81	9.82	16.71	16.08	12.18	21.22
25-26	294	70.0	126	30.0	12.23	9.92	15.07	14.27	11.42	17.82
27-28	468	73.6	168	26.4	10.24	8.58	12.23	11.45	9.48	13.83
29-30	770	79.8	195	20.2	7.22	6.17	8.46	8.07	6.82	9.54
31-32	1532	86.9	230	13.1	4.28	3.72	4.92	4.67	4.03	5.42
33-34	3948	88.6	506	11.4	3.66	3.33	4.02	3.76	3.41	4.16
35-36	14626	91.3	1393	8.7	2.72	2.57	2.88	2.68	2.52	2.84
37+	394483	96.6	13828	3.4	1+			1+		
*Sex*										
Male	215446	96.5	7781	3.5	1+			1+		
Female	200847	95.8	8743	4.2	1.21	1.17	1.24	1.22	1.18	1.26
*Small for gestational age*										
No	377022	96.4	14181	3.6	1+			1+		
Yes	39271	94.4	2343	5.6	1.59	1.52	1.66	1.34	1.27	1.40

*Total*	416297	96.2	16525	3.8						

In some cases the sum does not add up to the total because of missing values.

NE = Item risk not estimated because of collinearity with “previous deliveries” variable.

°Including all the above listed variables.

**Table 2:** Distribution and corresponding odds ratios of cases with gestational age ≥37 weeks, with vertex and breech presentation, according to selected factors. Lombardy, Italy.

	*Presentation*				*Unadjusted logistic regression*			*Adjusted logistic regression *∗		
	*Vertix*		*Breech*			*95% CI*			*95% CI*	
	*N*	*%*	*N*	*%*	*OR*	*Lower*	*Upper*	*OR*	*Lower*	*Upper*
*Maternal age*										
<25	41162	97.4	1113	2.6	1+			1+		
25–29	86512	97.0	2690	3.0	1.15	1.07	1.23	1.23	1.14	1.32
30–34	134076	96.5	4862	3.5	1.34	1.25	1.43	1.46	1.36	1.57
35–39	104283	96.3	3965	3.7	1.41	1.31	1.50	1.58	1.47	1.71
40+	27365	96.0	1155	4.0	1.56	1.44	1.70	1.62	1.48	1.78
*Maternal education*										
University	112538	96.4	4144	3.6	1+			1+		
High school degree	170611	96.5	6183	3.5	0.98	0.95	1.02	1.06	1.02	1.11
Primary/intermediate school or no education	109644	97.0	3446	3.0	0.85	0.82	0.89	1.07	1.01	1.12
*Citizenship*										
Italian	278611	96.4	10453	3.6	1+			1+		
Foreign	115872	97.2	3375	2.8	0.78	0.75	0.81	0.95	0.91	0.996
Albanian	10544	97.0	323	3.0	0.82	0.73	0.91			
Chinese	6021	97.7	140	2.3	0.62	0.52	0.73			
Egyptian	6855	97.6	166	2.4	0.65	0.55	0.75			
Moroccan	15508	97.5	393	2.5	0.68	0.61	0.75			
Romanian	13365	96.3	509	3.7	1.02	0.93	1.11			
Other	63579	97.2	1844	2.8	0.77	0.74	0.81			
*Previous delivery*										
0	198363	95.6	9065	4.4	1+			1+		
1	140420	97.6	3515	2.4	0.55	0.53	0.57	0.41	0.39	0.43
2+	48980	98.0	1010	2.0	0.45	0.42	0.48	0.35	0.33	0.38
Previous parity in unidentified number	6566	96.5	235	3.5	0.78	0.69	0.89	0.48	0.42	0.56
*Previous cesarean*										
Nulliparous	198363	95.6	9065	4.4	2.30	2.20	2.40	NE		
0	127389	98.0	2536	2.0	1+			1+		
1	35829	95.9	1530	4.1	2.15	2.01	2.29	1.41	1.31	1.50
2+	7132	97.0	221	3.0	1.56	1.35	1.79	0.96	0.82	1.11
*Previous spontaneous or induced abortion*										
No	279954	96.6	9990	3.4	1+			1+		
Yes	114529	96.8	3838	3.2	0.94	0.90	0.98	1.03	0.99	1.07
*PMA*										
Spontaneous conception	387371	96.7	13423	3.3	1+			1+		
Assisted conception	7112	94.6	405	5.4	1.64	1.48	1.82	1.03	0.93	1.14
FIVET	2234	95.1	116	4.9	1.50	1.24	1.81			
ICSI	2875	94.2	177	5.8	1.78	1.53	2.07			
Other	2003	94.7	112	5.3	1.61	1.33	1.95			
*Sex*										
Male	203560	97.0	6397	3.0	1+			1+		
Female	190920	96.3	7430	3.7	1.24	1.20	1.28	1.23	1.22	1.31
*SGA*										
No	382060	96.6	13290	3.4	1+			1+		
Yes	8294	95.1	423	4.9	1.54	1.47	1.62	1.25	1.19	1.32

*Total*	394483	96.6	13828	3.4						

In some cases the sum does not add up to the total because of missing values.

1+ Reference category

NE = Item risk not estimated because of collinearity with “previous deliveries” variable.

∗Multivariate logistic model including all the variables reported in the column.

## Data Availability

Data supporting the results reported in this article can not be found in public datasets as its owned by Regione Lombardia (The Health Authority).

## Abbreviations

ART: Assisted reproduction technology
CS: Cesarean section
SGA: Small for gestational age
IUGR: Intrauterine growth restriction
ECV: External cephalic version.
